# The Possible Role of Internet-Delivered Psychological Interventions in Relation to the COVID-19 Pandemic

**DOI:** 10.32872/cpe.v2i3.3941

**Published:** 2020-09-30

**Authors:** Gerhard Andersson, Matilda Berg, Heleen Riper, Jonathan D. Huppert, Nicolai Titov

**Affiliations:** a Department of Behaviorial Sciences and Learning, Linköping University, Linköping, Sweden; b Department of Clinical, Neuro and Developmental Psychology, Vrije Universiteit, Amsterdam, the Netherlands; c Department of Research and Innovation, GGZ in Geest/Amsterdam University Medical Center, VU University Medical Center, Amsterdam, the Netherlands; d The Hebrew University of Jerusalem, Mount Scopus, Jerusalem, Israel; e MindSpot Clinic, Macquarie University, Sydney, Australia; f eCentreClinic, Department of Psychology, Macquarie University, Sydney, Australia

The consequences of the COVID-19 pandemic are moving targets, making it hard to estimate the societal burden in terms of not only physical but also mental health (Holmes et al., 2020). It is clear that mental health problems will increase as a consequence of the pandemic. However, the specific problems across countries will reflect their response to the pandemic with mental health problems including the effects of social isolation (physical distancing), loss followed by disrupted grief ceremonies, loss or disruption to vocational, economic or educational opportunities, fear of a second outbreak of COVID-19 and future post-corona mental health consequences (Holmes et al., 2020). Recent studies indicate that service demands for psychiatric assessments and interventions have increased (Titov et al., 2020), while at the same time in person psychiatric visits for mild to moderate conditions have been advised against.

There are many new challenges and possibilities raised by the pandemic. It is likely that we will see new problems and new groups of clients not seen before. Mental health problems among health care workers is one example, and loneliness or relationship distress caused by social distancing is another example. A third example could be coping with loss: death of loved ones with little opportunity for social support, loss of employment and monetary loss, and loss or disruption to education. To our knowledge, with the possible exception of problem-solving therapy and interpersonal psychotherapy focused on bereavement and role change, few psychological treatment studies have targeted financial concerns and mental health problems in association with such changes. The lesson for researchers is to document and adapt according to the new situation.

Provision of evidence-based psychological treatments that not only are cost-effective but also safe to deliver from a pandemic perspective would have relied solely on telephone contacts before the advent of modern information technology (Wind, Rijkeboer, Andersson, & Riper, 2020). Since the late 1990s, a wide range of evidence-based internet interventions have been developed for a range of psychiatric diagnoses (for example major depression, anxiety and substance use disorders), and also psychological problems like loneliness, insomnia and stress (Andersson, Titov, Dear, Rozental, & Carlbring, 2019). Internet interventions often include instructions on how to perform tasks in real life. For example, exposure to feared social situations are performed in real life, and virtual reality and attention training may be used to augment or facilitate real life activities (Miloff, Lindner, & Carlbring, 2020). This leads to one immediate challenge in the era of COVID-19: homework assignments must be adapted to the current regulations and restrictions in each jurisdiction. Real-time video conferencing is a further alternative to deliver evidence-based psychological treatments (Varker, Brand, Ward, Terhaag, & Phelps, 2019). However, it is important to note that few studies have evaluated this treatment format and that it is more costly than internet interventions that involve minor therapist input.

In spite of the many advantages of internet interventions there are additional limitations that are specifically relevant in view of the pandemic: First, internet interventions are rarely used for clients with severe mental health problems (e.g., psychosis and acute suicidal intent) and therefore cannot be a total solution in providing remote access to mental health care. Second, with the COVID-19 pandemic there has been an increase in the use of video consultations. While it is likely that video therapy works as well as face-to-face therapy, this has not been tested in empirical studies to the same extent as internet interventions in the form of guided self-help (Varker et al., 2019). Third, although a decreasing proportion of the population continue to experience the digital divide, still far from all people in the world have access to reliable internet. Now, a majority have access, but it is still the case that there are groups who are not able to use computers or smartphones, including frail, older persons, persons with intellectual disabilities, or those socio-economically disadvantaged. As a fourth limitation we raise the risk of not performing proper diagnostic assessments as is standard practice in most clinical settings (e.g., primary care and also some clinics providing internet interventions), where patients are screened for general health. In other words, internet interventions benefit from a well-functioning health care in order to maintain not only good quality treatment but also ethical standards when referral is needed. For example, if a cardiac problem is suspected in a telephone interview it may be more difficult to refer the client to regular health care.

Despite these limitations, Internet interventions research has the advantage that treatments can be adapted rapidly and tested more quickly than is the case in regular psychotherapy research (and also medical research). There are several previous examples of this with treatments being developed for problems like loneliness, procrastination and perfectionism, but also adapting treatments for different age groups (e.g., adolescents, adults and older adults). Furthermore, one striking advantage of internet interventions is translation and cultural adaption of interventions that would be very hard to deliver using a translator or expensive when training therapists in new settings (Andersson et al., 2019). There are now studies on internet treatments in many languages including Arabic, Mandarin, and Hebrew just to give a few examples. Given the limited resources in many places and the risk of even worse economic circumstances, there is need and opportunity to develop and test interventions that are accessible regardless of where the person resides. Of course, it is crucial that the medico-legal and clinical aspects are carefully managed, but this is a likely development in the future.

In conclusion, the current COVID-19 pandemic situation does not allow us to wait. Internet-delivered psychological interventions should be offered and in particular evidence-based Internet interventions that allow privacy and can be adapted for different problems and languages. Specific interventions for psychological problems related to COVID-19 should be developed. This could help reduce the societal burden caused by the pandemic.
